# Oculomotor fatigability with decrements of saccade and smooth pursuit for diagnosis of myasthenia gravis

**DOI:** 10.1007/s00415-023-11611-7

**Published:** 2023-03-01

**Authors:** Thanh Tin Nguyen, Jin-Ju Kang, Ju-Hee Chae, Eunsu Lee, Hyo-Jeong Kim, Ji-Soo Kim, Sun-Young Oh

**Affiliations:** 1 Department of Neurology, Jeonbuk National University Hospital, Jeonbuk National University School of Medicine, Jeonju, South Korea; 2 Research Institute of Clinical Medicine of Jeonbuk National University-Biomedical Research Institute of Jeonbuk National University Hospital, Jeonju, South Korea; 3 Department of Pharmacology, Hue University of Medicine and Pharmacy, Hue University, Hue, Vietnam; 4 Department of Neurology, Seoul National University, Seoul National University Bundang Hospital, Seongnam, South Korea

**Keywords:** Myasthenia gravis, Ocular myasthenia gravis, Generalized myasthenia gravis, Video-oculography, Saccade, Smooth pursuit, Fatigability, Oculomotor fatigability

## Abstract

**Background and objectives:**

As the efficacy of current diagnostic methods for myasthenia gravis (MG) remains suboptimal, there is ongoing interest in developing more effective diagnostic models. As oculomotor fatigability is one of the most common and diagnostic symptoms in MG, we aimed to investigate whether quantitative saccadic and smooth-pursuit fatigability analyses with video-oculography (VOG) are useful for diagnosis of MG.

**Methods:**

A convenience cohort of 46 MG patients was recruited prospectively, including 35 with ocular and 11 with generalized MG (mean age, 50.9 ± 14.5 years; 17 females); 24 healthy controls (HCs) (mean age, 50.6 ± 16.3 years; 13 females) also were enrolled. Seventy-five repetitive saccades and smooth pursuits were recorded in ranges of 20° (horizontal plane) and 15° (vertical plane) using a three-dimensional VOG system. Based on the oculomotor range of the second saccade and smooth pursuit and the mean ranges of the last five of each, the estimated decrements (%) reflecting oculomotor fatigability were calculated.

**Results:**

The baseline oculomotor ranges did not show significant difference between the MG and HCs groups. However, following repetitive saccades and pursuits, the oculomotor ranges were decreased substantially during the last five cycles compared to baseline in the MG group. No such decrements were observed in the HC group (*p* < 0.01, Mann–Whitney *U* test). Receiver operating characteristic (ROC) analysis revealed that repetitive vertical saccades yielded the best differentiation between the MG and HC groups, with a sensitivity of 78.3% and specificity of 95.8% when using a decrement with an amplitude of 6.4% as the cutoff.

**Conclusion:**

This study presents an objective and reproducible method for measuring decrements of oculomotor ranges after repetitive saccadic and pursuit movements. Quantification of oculomotor fatigability using VOG could be a sensitive and specific diagnostic tool for MG and allows easy, cost-effective, accurate, and non-invasive measurements.

**Classification of evidence:**

This study provides class III evidence that VOG-based quantification of saccadic and pursuit fatigability accurately identifies patients with MG.

## Introduction

Myasthenia gravis (MG) is an autoimmune disorder characterized by fatigability and fluctuating muscle weakness induced by auto-antibodies binding to the postsynaptic region at the neuromuscular junction (NMJ) [1–4]. Specific auto-antibody formations against acetylcholine receptors (AChRs), muscle-specific kinase (MuSK), or lipoprotein-related peptide 4 [1, 5–11] lead to transmission deficits at the NMJ and its eventual destruction [1, 11, 12]. Extraocular muscles are particularly susceptible to dysfunction, and ocular weakness presenting as ptosis or diplopia is the most common initial presentation of MG. This ocular form of MG (OMG) (15%) in which the weakness is limited to the extraocular, levator, and orbicularis oculi muscles progresses to a generalized form of MG (GMG) (85%), in which the weakness is generalized to involve the limb, bulbar, or respiratory musculature in about 50% of cases, usually within 2 or 3 years [13–15]. Diagnosis of OMG can be challenging due to symptomatic variability and vague diagnostic criteria in seronegative patients, as well as insufficient sensitivity of current diagnostic methods. Unfortunately, conventional diagnostic tests for OMG have not demonstrated satisfactory sensitivity, specificity, and cost-effectiveness [16, 17]. Single-fiber electromyography has higher sensitivity in detecting OMG (62–97%) [18] compared to other ancillary tests, but is time-consuming and requires a skilled neurophysiologist [2, 18]. Therefore, to eliminate clinical ambiguity and approach a correct diagnosis of MG [19–21], many supportive laboratory techniques have been introduced, such as repetitive ocular vestibular evoked myogenic potential (oVEMP) test. Specifically, repetitive oVEMP tests evaluate decrements in ocular muscle activity of MG patients, where a unilateral decrement ≥ 15.2% yielded a sensitivity of 89% and specificity of 64% [22, 23]. One drawback of oVEMP testing is that any structural or functional lesion present between the otoliths and extraocular muscles affecting the vestibulo-ocular reflex (VOR) pathways can affect the oVEMP amplitude.

Although several previous studies have examined oculomotor fatigability in normal subjects and MG patients [24–26], their results have not been validated or translated into clinical practice for application of quantitative oculomotor fatigability for diagnosing MG [24–26]. With the development of techniques for recording and analyzing eye movements, oculomotor fatigability could be more easily evaluated and thoroughly described [24–28]. In the current prospective case–control study, we aimed to investigate the diagnostic yield of quantitation of oculomotor fatigability based on the decrement of oculomotor ranges after repetitive saccades and smooth pursuits using three-dimensional video-oculography (VOG) to distinguish MG patients from healthy controls (HCs).

## Methods

### Participants

We included a convenience cohort of MG patients who visited Jeonbuk National University Hospital between April 2022 and November 2022 (*n = *46; mean age, 50.93 ± 14.53 years; age range, 18–81 years; 17 females) (Table 1). Diagnosis of MG was based on the presence of a typical clinical history validated by a senior neurologist (S.Y Oh), ≥ 1 positive ancillary test including repetitive nerve stimulation (RNS) and edrophonium tests, and serum auto-antibodies against AChRs or MuSK [19, 29, 30]. AChR antibody seronegative patients were tested with serum auto-antibodies against MuSK. Patients with myasthenic symptoms but negative results from traditional investigations, drug-induced myasthenia, or Lambert–Eaton myasthenic syndrome were excluded. For comparison, we included 24 age- and sex-matched healthy controls (HCs) (mean age, 50.58 ± 16.28 years; age range, 23–81 years; 13 females) without neuromuscular, vestibular, or oculomotor disorders (Table 1). We classified the patients into an ocular-onset and isolated ocular MG (OMG) group in which patients presented with pure ocular symptoms such as ptosis or diplopia and a generalized MG (GMG) group in which patients experienced weakness of the limbs or the facial, bulbar, neck, or respiratory muscles.

**Table 1:** Demographic and clinical features of MG patients and HCs

	Myasthenia gravis (*n = *46)				HC (*n = *24)	*P* value^M^ (MG vs. HC)
	Ocular MG (*n = *35, 76%)	Generalized MG (*n = *11, 24%)	Total (*n = *46)	*P* value (OMG vs. GMG)		
Demographics						
Sex, female (%)	13 (37.1)	4 (36.4)	17 (37)	0.963	13 (54.2)	0.207
Age, mean ± SD, years	51.26 ± 14.30	49.91 ± 15.90	50.93 ± 14.53	0.671	50.58 ± 16.28	0.724
Disease duration, median (95% CI), months	45.4 (1.2–94.5)	52.5 (4.93–115.17)	16.48 (2.2–52.5)	0.309		
Clinical presentations						
Ptosis (%)	33 (94.3)	5 (45.5)	38 (82.6)	** < 0.001**	–	–
Diplopia (%)	32 (91.4)	9 (81.8)	41 (89.1)	0.377	–	–
Dysarthria (%)	–	4 (36.4)	4 (8.7)		–	–
Dysphagia (%)	–	2 (18.2)	2 (4.3)		–	–
Dyspnea (%)	–	3 (27.3)	3 (6.5)		–	–
Motor weakness (%)	–	9 (81.8)	9 (19.6)		–	–
Thymoma (%)	6 (17.1)	0 (0)	6 (13.0)	0.145	–	–
Thymic hyperplasia (%)	6 (17.1)	3 (27.3)	9 (19.6)	0.111	–	–
Ancillary tests						
Acetylcholine receptor Ab, positive (%)	21 (60)	11 (100)	32 (69.6)	**0.013**	–	–
Repetitive nerve stimulation decrement, positive (%)	11 (31.4)	8 (72.7)	19 (41.3)	**0.016**	–	–
Edrophonium test, positive (%)	16 (45.7)	9 (81.8)	25 (54.3)	**0.038**	–	–
Treatments						
Choline esterase inhibitor (pyridostigmine)	34 (97.1)	10 (90.9)	44 (95.7)	0.377	–	–
Steroid	15 (42.8)	6 (54.5)	21 (45.7)	0.478	–	–
Immunosuppressants	21 (60)	8 (72.7)	29 (63)	0.446	–	–
Thymectomy	5 (14.6)	4 (36.4)	9 (19.6)	0.254	–	–

Statistical significance was calculated using Mann–Whitney *U* test. All tests were performed at a 0.05 level of significance

*Ab* antibody, *CI* confidence interval; *HC* healthy control; *M* Mann–Whitney *U* test; *MG* myasthenia gravis; *SD* standard deviation

### Standard protocol approvals, registrations, and patient consent

All participants provided informed consent and received monetary compensation for participation. Experiments were reviewed and approved by the Institutional Review Board at Jeonbuk National University Hospital (no. 2022-04-044-001).

### Video-oculography recordings

Participants were comfortably seated in a dimly lit room, with their heads stabilized against the chair’s headrest, and were given directions regarding each test. Participants were asked not to ingest caffeine-containing foods, alcohol, or acetylcholinesterase inhibitors for 24 h or corticosteroids for 72 h before the experimental session [31–33]. Oculomotor recordings were conducted in the morning between 9 and 11 o’clock after having breakfast. Participants’ eye movements were tracked using computerized recording equipment of three-dimensional VOG (SMI, Teltow, Germany) with a resolution of 0.1° and sampling rate of 60 Hz in a completely dark room. Stimuli were presented using custom-made software with a fixation cue positioned in the center of the visual field at a viewing distance of 1.5 m. Participants' heads were restrained using foam-lined clamps placed on either side of the head and at the chin. A bandage was applied to the eyelids of the blepharoptosis patients, and eye movements were recorded. The targets used during calibration and testing were red light-emitting diodes that subtended to 0.29° × 0.58° of the visual field. Each subject was instructed to follow the target as accurately as possible. The eye-tracking software filtered and excluded deviations from the target (e.g., blinks) or saccades occurring during pursuit tasks. Digitized data were analyzed using Matlab software (version R2022b; MathWorks Inc., Natick, MA, USA).

**Fig. 1:**
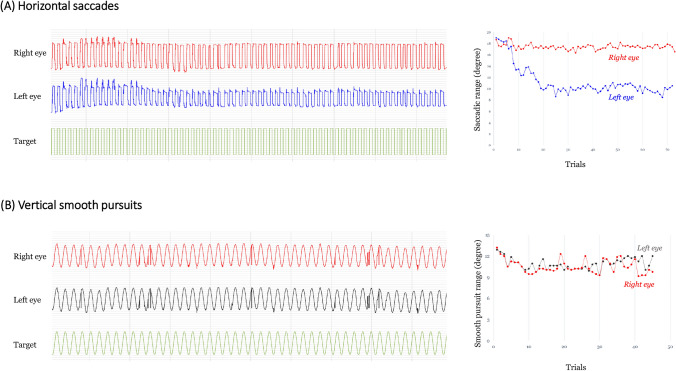
Repetitive oculomotor recordings from both eyes of an MG patient. **A** Representation of the decrement of saccadic range in the left eye during repetitive 20° horizontal saccades and **B** decrements of smooth-pursuit ranges during 15° vertical smooth pursuits of both eyes

After a calibration task, saccades were recorded from both eyes in ranges of ± 20° (horizontal plane) and ± 15° (vertical plane) (Fig. 1). For horizontal saccades, successive targets regularly appeared for a presentation time of 1 s alternatively from right to left or left to right at a rate of 15 saccades/min (0.25 Hz) for 5 min (total 75 cycles). Similarly, for vertical saccades, successive targets regularly appeared for 1 s alternatively from up to down or down to up for 75 cycles. There were no intervals of successive positions between targets. During horizontal smooth pursuit (SP), the target moved smoothly across the display at 20° gaze from midline alternately from right to left or left to right for 75 cycles. During vertical smooth pursuits, the target moved smoothly up to down across the display at 15° gaze from a horizontal line. The target moved at a speed of 10°/s in a predictable, oscillating sinusoidal waveform. To reduce fatigability derived from the cumulative effect of serial trials of horizontal and vertical saccades and pursuits, 3-min rest intervals were allowed between each trial.

The average decrement of the oculomotor range (peak-to-peak amplitude) of the last five saccades/smooth pursuits compared to that of the second saccade/smooth pursuit was analyzed. The first response of saccades and smooth pursuits showed high variability in amplitude change, even in healthy controls. Therefore, we used the second response of saccade and smooth pursuit as the reference and defined decrement as the difference between the second oculomotor response and the average of the last five responses. As a measure of neuromuscular transmission and fatigability, the decrements of oculomotor range after repetitive saccades or pursuits were estimated for each eye based on a previous description [22, 23] using the following formula:

$${\text{Decrement of the saccadic range}} = 100\% - \frac{{{\text{ average of the last }}5{\text{ saccadic ranges}}}}{{\text{the second saccadic range}}} \times 100 \% ,$$

$${\text{Decrement of the SP range}} = 100\% - \frac{{{\text{ average of the last }}5{\text{ smooth}} - {\text{pursuit ranges}}}}{{{\text{ the second smooth}} - {\text{pursuit range}}}} \times 100 \% .$$

Figure 1 illustrates oculomotor recordings from an MG patient, illustrating the decrements of the oculomotor ranges.

### Statistical analysis

Data were processed and analyzed using MATLAB version 9.13 (R2022b; MathWorks Inc., Natick, MA, USA) and SPSS Statistics version 23.0 (IBM Corp., Armonk, NY, USA). Non-parametric variables are presented as median (95% confidence interval [CI]). The Mann–Whitney *U* test and Pearson's Chi-square test were used to compare differences between the MG and HC groups and between ocular and generalized MG subgroups. Receiver operating characteristic (ROC) curve analysis was applied to determine the area under the ROC curve (AUC), sensitivity, specificity, likelihood ratio (LR), predictive values, cutoff value, and significance of the optimal decrement for each VOG parameter. Correlations between the oculomotor range decrements and clinical features were assessed using Spearman's non-parametric bivariate correlation. All tests were performed at a 0.05 level of significance.

## Results

### Clinical characteristics

The demographic and clinical characteristics of 46 patients with MG are depicted in Table 1. Thirty-five patients had OMG (35/46, 76%) and 11 patients had GMG (11/46, 24%). There were no significant differences in sex, age, and disease duration between the ocular and generalized MG subgroups. Mean age at diagnosis of OMG was 44.9 years (SD 12.1) and that at inclusion was 51.3 years (SD 14.3). In comparison, the mean age at diagnosis of GMG was 45.5 years (SD 22.4) and that at inclusion was 49.9 years (SD 15.9) (Table 1). Ptosis was more common in OMG patients than GMG patients (94.3% *vs*. 45.5%; *p* < 0.001, Mann–Whitney *U* test), but rates of diplopia symptoms were similar between the groups. Other generalized symptoms, such as limbs weakness (81.8%), dysarthria (36.4%), dysphagia (18.2%), and dyspnea (27.3%), were reported frequently during the prior 1 month in the GMG group. Ancillary diagnostic tests were evaluated, and the serum AChR antibody result was positive in 69.6% of MG patients (32/46; 60% for OMG patients and 100% for GMG patients; *p* = 0.013, Mann–Whitney *U* test). The RNS result was positive in 41.3% of total MG patients (19/46; 31.4% for OMG patients and 72.7% for GMG patients; *p* = 0.016, Mann–Whitney *U* test), and the edrophonium test result was positive in 58.7% (25/46; 45.7% for OMG patients and 81.8% for GMG patients; *p* = 0.038, Mann–Whitney *U* test) of MG patients. AChR antibody seronegative patients were tested with serum auto-antibodies against MuSK by radioimmunological assay and all were negative.

After diagnosis of MG, 95.7% (44/46) of patients were taking a choline esterase inhibitor (pyridostigmine) at a mean dosage of 218.18 ± 82.95 mg/day (215.29 ± 72.54 mg/day in the OMG subgroup and 228.00 ± 115.93 mg/day in the GMG subgroup). Twenty-one patients (21/46, 45.7%) received additional steroid therapies with a mean dosage of 9.29 ± 3.96 mg/day (8.82 ± 0.81 mg/day in the OMG subgroup and 11.25 ± 6.29 mg/day in the GMG subgroup), while 29 patients (29/46, 63.0%) received additional immunosuppressant therapies, such as tacrolimus (16 cases), azathioprine (10 cases), or mycophenolate (3 cases). Thymectomy was performed in 9 patients (19.6%) between 6 months and 5 years after diagnosis of MG (Table 1).

### Oculomotor range decrements after repetitive saccades and smooth pursuits in MG patients

**Fig. 2:**
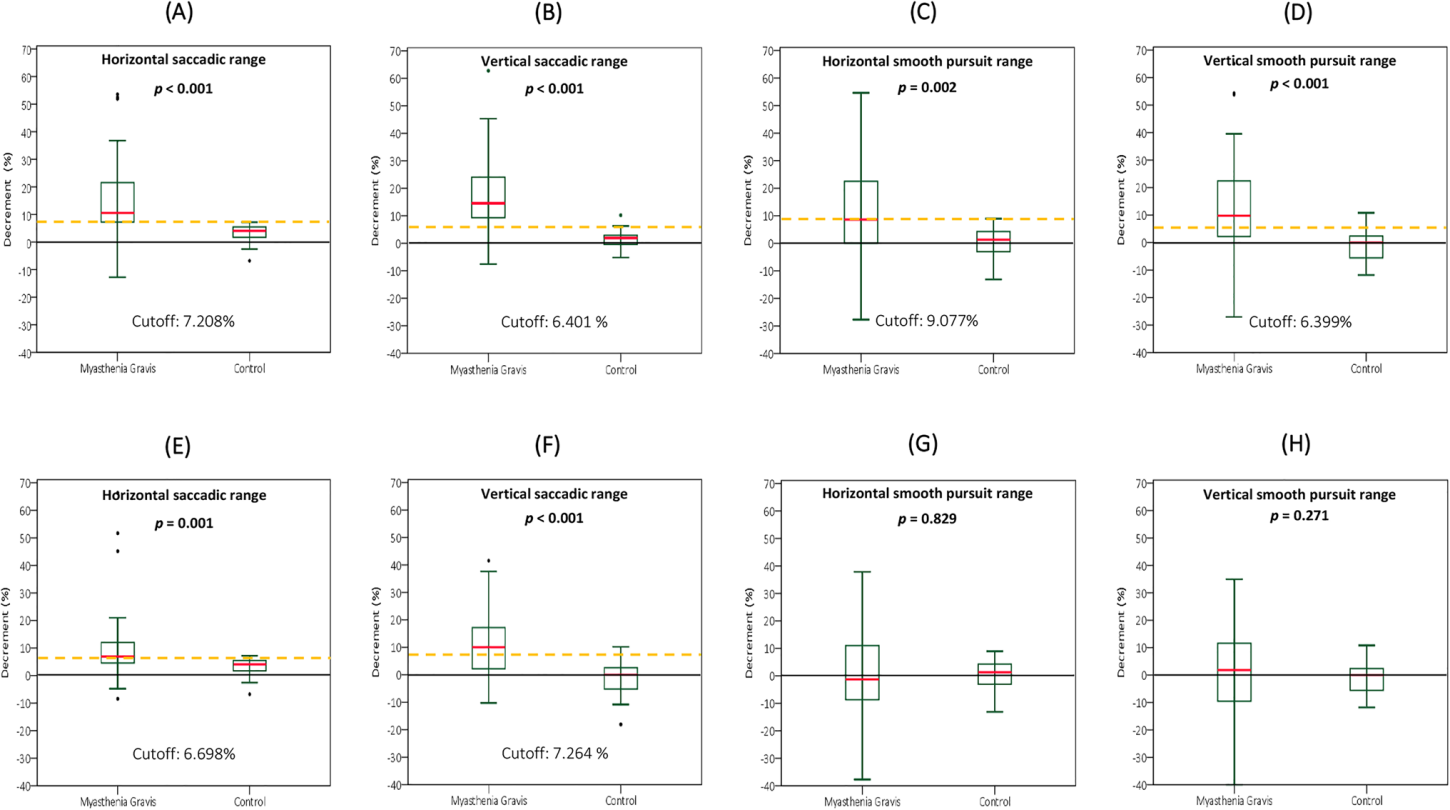
Comparison of the decrements of oculomotor ranges in MG patients and HCs in the more-affected eyes (**A**–**D**) and in the less-affected eyes (**E**–**H**). The dotted yellow lines indicate optimal diagnostic cutoffs for decrements of horizontal **A** and vertical **B** saccades and horizontal (**C**) and vertical (**D**) smooth pursuits in the more-affected eyes and of horizontal (**E**) and vertical (**F**) saccades and horizontal (**G**) and vertical (**H**) smooth pursuits in the less-affected eyes. The boxes show the median (red line) and first and third quartiles, while the ends of the whiskers represent the most extreme data points excluding outliers. The decrements of saccadic and pursuit ranges in MG patients were larger in both the more-affected eye and the less-affected eye compared to those in HCs

Figure 1A illustrates repetitive saccadic recordings from an MG patient who showed a striking decrement of saccadic range in the left eye (the more-affected eye) starting at about the 10th saccade. The range of the second saccadic recording did not differ between the MG and HC groups during either horizontal (*p* > 0.05, Mann–Whitney *U* test) or vertical (*p* > 0.05) saccades (Table 2). However, together with repetitive saccadic eye movements, in the horizontal plane, the decrement of the saccadic range in MG patients was larger in both the more-affected eye (11.92%; *p* < 0.001, Mann–Whitney *U* test) and the less-affected eye (7.07%; *p* = 0.001, Mann–Whitney *U* test) than it was in the HC group (4.3%) (Table 2 and Fig. 2A and E). Similarly, in the vertical plane, the estimated decrement of the saccadic range in MG patients was larger in both the more-affected eye (14.63%; *p* < 0.001, Mann–Whitney *U* test) and the less-affected eye (13.48%; *p* < 0.001, Mann–Whitney *U* test) than in the HC group (2.545%) (Table 2 and Fig. 2B and F).

**Table 2:** Oculomotor fatigabilities of MG patients and HCs

	Myasthenia gravis				Control (*n = *24)	*P* value^M^
	Ocular MG (*n = *35)	Generalized MG (*n = *11)	Total (*n = *46)	*P* value^M^		
Saccadic eye movement, horizontal						
Oculomotor ranges, more-affected eye						
Second saccade, median (95% CI)	19.6 (19–20)	19.6 (9.4–20.6)	19.6 (19–19.8)	0.462	19.75 (18.9–20.3)	0.674
Last 5 saccades, median (95% CI)	17 (16.2–18.2)	16.6 (7.6–18.4)	17 (16–18.2)	0.315	19 (18.7–19.4)	** < 0.001**
Decrement %, median (95% CI)	10.31 (8.41–17.35)	16.78 (7.84–31.63)	11.92 (8.79–17.35)	0.511	4.3 (2.31–5.56)	** < 0.001**
Oculomotor ranges, less-affected eye						
Second saccade, median (95% CI)	19.2 (18.6–19.6)	19.6 (15–21)	19.3 (18.6–19.8)	0.969	19.75 (18.9–20.3)	0.353
Last 5 saccades, median (95% CI)	18 (17.6–18.6)	16.8 (13.2–19)	17.9 (17.2–18.4)	0.315	19 (18.7–19.4)	**0.004**
Decrement %, median (95% CI)	6.8 (5.21–10.96)	10 (5.71–16.67)	7.07 (6.12–10.96)	0.092	4.3 (2.31–5.56)	**0.001**
Saccadic eye movement, vertical						
Oculomotor ranges, more-affected eye						
Second saccade, median (95% CI)	13.8 (13.35–14.55)	12.9 (8.7–15)	13.65 (13.2–14.4)	0.116	14.065 (13.65–14.4)	0.432
Last 5 saccades, median (95% CI)	11.85 (10.95–12.6)	11.55 (6.15–12.9)	11.78 (10.95–12.5)	0.296	13.615 (13.28–14.33)	** < 0.001**
Decrement %, median (95% CI)	14.76 (11.46–22.43)	12.24 (5.88–41.76)	14.63 (11.46–21.6)	1	2.545 (1.89–4.9)	** < 0.001**
Oculomotor ranges, less-affected eye						
Second saccade, median (95% CI)	13.8 (13.2–14.4)	13.5 (8.55–14.4)	13.73 (13.05–14.1)	0.353	14.065 (13.65–14.4)	0.202
Last 5 saccades, median (95% CI)	12.15 (11.55–13.05)	12.3 (6.75–12.75)	12.23 (11.55–12.9)	0.268	13.615 (13.28–14.33)	** < 0.001**
Decrement %, median (95% CI)	14.13 (9.3–17.35)	10.38 (2.3–34.04)	13.48 (9.3–16.04)	0.777	2.545 (1.89–4.9)	** < 0.001**
Smooth pursuit, horizontal						
Oculomotor ranges, more-affected eye						
Second smooth pursuit, median (95% CI)	19.58 (16.5–22.22)	19.14 (10.56–28.82)	19.36 (16.5–21.78)	0.847	20 (19.1–22.2)	0.443
Last 5 smooth pursuits, median (95% CI)	16.06 (14.74–18.42)	15.18 (7.04–25.08)	16.1 (14.74–18.92)	0.709	19.95 (18.2–21.9)	**0.008**
Decrement %, median (95% CI)	13.67 (6.49–24.17)	11.19 (0–42.86)	13.67 (7.13–20)	0.690	3.43 (1.48–6.19)	**0.002**
Oculomotor ranges, less-affected eye						
Second smooth pursuit, median (95% CI)	17.82 (15.62–22)	17.6 (8.8–26.18)	17.71 (15.62–21.56)	0.495	20 (19.1–22.2)	0.102
Last 5 smooth pursuits, median (95% CI)	16.94 (15.84–19.58)	16.72 (9.68–26.18)	16.83 (15.6–19.58)	0.738	19.95 (18.2–21.9)	**0.028**
Decrement %, median (95% CI)	11.93 (6.32–20.99)	6.38 (2.5–36.13)	11.47 (5.94–22.5)	0.634	3.43 (1.48–6.19)	0.829
Smooth pursuit, vertical						
Oculomotor ranges, more-affected eye						
Second smooth pursuit, median (95% CI)	14.7 (11.55–16.05)	16.2 (9.45–18.3)	15.07 (11.55–16.2)	0.479	15.26 (14.25–16.58)	0.512
Last 5 smooth pursuits, median (95% CI)	12.6 (10.35–14.55)	14.4 (5.1–16.95)	13.05 (10.95–14.7)	0.471	15.34 (14.55–16.8)	**0.002**
Decrement %, median (95% CI)	14.29 (8.41–23.02)	16.67 (0.99–28.99)	14.28 (8.45–20.4)	0.598	1.94 (0–5.71)	** < 0.001**
Oculomotor ranges, less-affected eye						
Second smooth pursuit, median (95% CI)	13.5 (10.35–15.15)	15.6 (6.15–17.55)	14.48 (10.5–15.3)	0.432	15.26 (14.25–16.58)	0.106
Last 5 smooth pursuits, median (95% CI)	13.95 (11.55–14.85)	15.6 (4.2–16.8)	14.18 (12.15–15.15)	0.334	15.34 (14.55–16.8)	**0.019**
Decrement %, median (CI)	9 (5–15.24)	8.73 (0.95–31.7)	9.01 (4.48–14.71)	0.374	1.94 (0–5.71)	0.271

Statistical significance was calculated using Mann–Whitney *U* test. All tests were performed at a 0.05 level of significance

*CI* confidence interval; *M* Mann–Whitney *U* test; *MG* myasthenia gravis

Comparing decrements of saccadic range between the OMG and GMG subgroups, no significant difference was revealed in either the horizontal plane (for more-affected eye: 10.31% *vs.* 16.78%; *p* = 0.511, for less-affected eye: 6.8% *vs.* 10%; *p* = 0.092, Mann–Whitney *U* test) or the vertical plane (for more-affected eye: 14.76% *vs.* 12.24%; *p* = 1, for less-affected eye: 14.13% *vs.* 10.38%; *p* = 0.777, Mann–Whitney *U* test) (Table 2).

There were also decrements of the smooth-pursuit range after repetitive pursuits in MG patients in both the horizontal and vertical planes. Figure 1B shows smooth-pursuit recordings from an MG patient showing a decrement in the smooth-pursuit range after about the 10th pursuit. The baseline (second) smooth-pursuit range did not differ between MG patients and HCs during horizontal (*p* > 0.05, Mann–Whitney *U* test) or vertical (*p* > 0.05) pursuits (Table 2). After repetitive pursuit movements, the estimated decrement of the pursuit range in the MG patients was significantly greater than that in the HC group in the more-affected eye in both the horizontal (13.67% *vs.* 3.43%; *p* = 0.002) and vertical (14.28% *vs.* 1.94%; *p* < 0.001, Mann–Whitney *U* test) planes (Table 2 and Fig. 2C and D). In the less-affected eyes, the mean ranges of the last five horizontal and vertical pursuits were significantly lower than those in the HC group; however, the decrements of the pursuit range did not differ from those in the HC group (Table 2 and Fig. 2G and H).

Comparing the decrements of the pursuit range between the OMG and GMG subgroups, there was no significant difference in either the horizontal plane (for the more-affected eye, 13.67% *vs.* 11.19%; *p* = 0.69, for the less-affected eye, 11.93% *vs.* 6.38%; *p* = 0.634, Mann–Whitney *U* test) or the vertical plane [for the more-affected eye, 14.28% *vs.* 16.67%; *p* = 0.598, for the less-affected eye, 9% *vs.* 8.73%; *p* = 0.374, Mann–Whitney *U* test] (Table 2).

### Cutoff values of saccadic and smooth-pursuit decrements for diagnosing MG

**Fig. 3:**
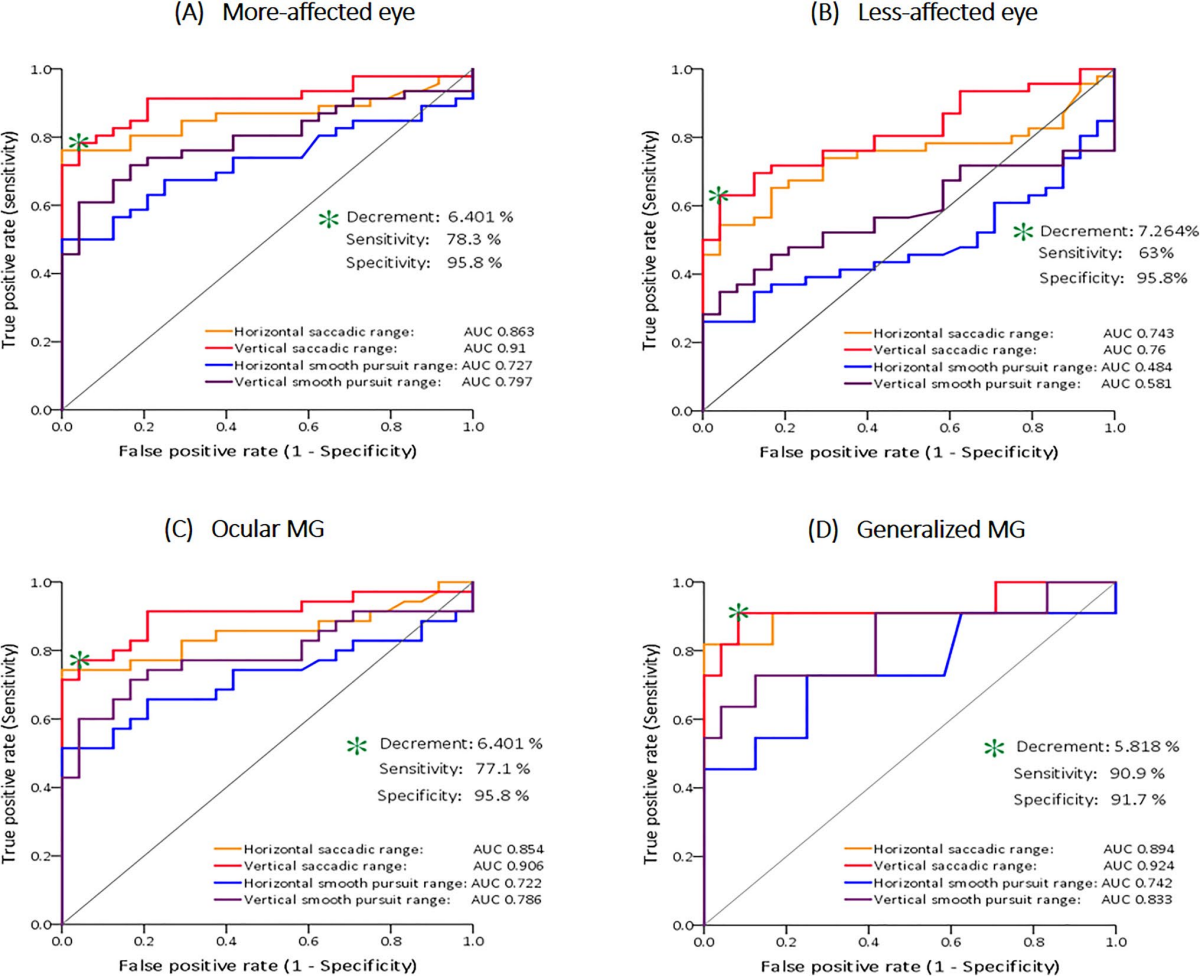
ROC curve analyses to determine optimal cutoffs for distinguishing MG patients. For both the more-affected eyes (**A**) and the less-affected eyes (**B**), AUC analysis showed that decrement of the saccadic range seems to provide greater discriminatory power than smooth-pursuit decrements despite their similar best cutoff values in ROC curve analysis. The green asterisk indicates the best cutoff value. For the more-affected eye, a vertical saccadic amplitude decrement ≥ 6.401% carries the advantage of a high sensitivity of 78.3% and a specificity of 95.8% (**A**). For the less-affected eye, a vertical saccadic amplitude decrement ≥ 7.264% carries the advantage of a high sensitivity of 63% and a specificity of 95.8% (**B**). For both ocular (**C**) and generalized (**D**) MG, the AUC was larger for vertical (red) than for horizontal (orange) saccades and vertical (blue) and horizontal (purple) smooth pursuits. For ocular MG, a vertical saccadic amplitude decrement ≥ 6.401% carries the advantage of a high sensitivity of 77.1% and a specificity of 95.8% (**C**). For generalized MG, a vertical saccadic amplitude decrement ≥ 5.818% carries the advantage of a high sensitivity of 90.9% and a specificity of 91.7% (**D**)

ROC curve analyses were applied to determine optimal cutoffs for distinguishing MG patients from HCs based on oculomotor decrements (Table 3 and Fig. 3). The AUC showed the decrements of saccadic ranges to detect myasthenia better than those of the smooth pursuits in both more- and less-affected eyes. For the more-affected eyes, the AUC of the horizontal saccades was o.863 (95% CI, 0.776–0.95; *p* < 0.001; best cutoff value, 7.208%; sensitivity, 76.1%; specificity, 100%), while that of the vertical saccades was 0.91 (95% CI, 0.841–0.979; *p* < 0.001; best cutoff value, 6.401%; sensitivity, 78.3%; specificity, 95.8%) (Table 3 and Fig. 3A). Similarly, for the less-affected eye, the AUC of the horizontal saccades was o.743 (95% CI, 0.629–0.857; *p* = 0.001; best cutoff value, 6.698%; sensitivity, 54.3%; specificity, 95.8%), while that of the vertical saccades was 0.76 (95% CI, 0.648–0.872; *p* < 0.001; best cutoff value, 7.264%; sensitivity, 63%; specificity, 95.8%) (Table 3 and Fig. 3B). The AUC for the horizontal smooth pursuits was o.727 (95% CI, 0.611–0.842; *p* = 0.002; best cutoff value, 9.077%; sensitivity, 50%; specificity, 100%), and that for the vertical smooth pursuits was 0.797 (95% CI, 0.694–0.9; *p* < 0.001; best cutoff value, 6.399%; sensitivity, 60.9%; specificity, 95.8%) in more-affected eyes. However, in less-affected eyes, the ROC analysis for smooth pursuits did not reveal a significant AUC value to differentiate MG patients from HCs in either the horizontal (*p* = 0.829) or vertical (*p* < 0.271) plane (Table 3 and Fig. 3A and B).

**Table 3:** Cutoff values and AUCs for MG diagnosis

	Cutoff values (decrement %)	Sensitivity, % (95% CI)	Specificity, % (95% CI)	LR (+)	LR (−)	PPV, % (95% CI)	NPV, % (95% CI)	AUC (95% CI)	*P* value
In more-affected eyes									
Saccadic range decrement, H	7.208	76.1 (60.9–86.92)	100 (82.83–100)	+ ∞	0.24	100 (87.68–100)	68.57 (50.58–82.57)	0.863 (0.776–0.95)	** < 0.001**
Saccadic range decrement, V	6.401	78.3 (63.24–88.55)	95.8 (76.88–99.78)	18.78	0.23	97.29 (84.19–99.59)	69.7 (51.13–83.79)	0.91 (0.841–0.979)	** < 0.001**
Smooth-pursuit range decrement, H	9.077	50 (35.12–64.88)	100 (82.83–100)	+ ∞	0.5	100 (82.19–100)	51.06 (36.25–65.7)	0.727 (0.611–0.842)	**0.002**
Smooth-pursuit range decrement, V	6.399	60.9 (45.39–74.54)	95.8 (76.88–99.78)	14.5	0.41	96.55 (80.37–99.82)	56.1 (39.89–71.18)	0.797 (0.694–0.9)	** < 0.001**
In less-affected eyes									
Saccadic range decrement, H	6.698	54.3 (39.15–68.82)	95.8 (76.88–99.78)	12.93	0.48	96.15 (78.42–99.8)	52.27 (36.88–67.27)	0.743 (0.629–0.857)	**0.001**
Saccadic range decrement, V	7.264	63 (47.52–76.4)	95.8 (76.88–99.78)	15	0.39	96.67 (80.95–99.82)	57.5 (41.01–72.57)	0.76 (0.648–0.872)	** < 0.001**
Smooth-pursuit range decrement, H	9.98	24.9 (13.08–39.1)	100 (82.83–100)	+ ∞	0.74	100 (67.85–100)	40.68 (28.33–54.24)	0.484 (0.351–0.618)	0.829
Smooth-pursuit range decrement, V	6.623	34.8 (21.77–50.32)	95.8 (76.88–99.78)	8.28	0.68	94.12 (69.24–99.69)	43.4 (30.1–57.64)	0.581 (0.45–0.711)	0.271

Statistical significance was calculated using ROC analysis at the significance level of 0.05

*AUC* area under the receiver operating characteristic curve; *CI* confidence interval; *H* horizontal; *LR* likelihood ratio; *MG* myasthenia gravis; *NPV* negative predictive value; *PPV* positive predictive value; *V* vertical

Additional analyses were carried out to investigate the cutoff values of oculomotor decrements for each ocular and generalized MG patient compared to HCs (Table 4 and Fig. 3C and D). For the more-affected eye, the saccadic cutoff values for the OMG subgroup were 7.208% (sensitivity, 74.3%; specificity, 100%; *p* < 0.001) for horizontal saccades and 6.40% (sensitivity, 77.1%; specificity, 95.8%; *p* < 0.001) for vertical saccades. Similarly, the pursuit cutoff values were 9.08% (sensitivity, 51.4%; specificity, 100%; *p* = 0.004) for horizontal smooth pursuits and 6.49% (sensitivity, 60%; specificity, 95.8%; *p* < 0.001) for vertical smooth pursuits (Table 4 and Fig. 3C). Meanwhile, the cutoff values for the GMG subgroup were 7.52% (sensitivity, 81.8%; specificity, 100%; *p* < 0.001) for horizontal saccades and 5.818% (sensitivity, 90.9%; specificity, 91.7%; *p* < 0.001) for vertical saccades. Similarly, the smooth-pursuit cutoff values were 3.88% (sensitivity, 72.7%; specificity, 75%; *p* = 0.023) in the horizontal plane and 5.00% (sensitivity, 72.7%; specificity, 87.5%; *p* = 0.002) in the vertical plane (Table 4 and Fig. 3D).

**Table 4:** Cutoff values and AUCs for diagnosing patients with ocular and generalized MG

	Cutoff values (decrement %)	Sensitivity (%)	Specificity (%)	LR ( +)	LR ( −)	AUC (95% CI)	*P* value
Ocular MG							
In more-affected eyes							
Saccadic range decrement, H	7.208	74.3	100	+ ∞	0.26	0.854 (0.753–0.954)	** < 0.001**
Saccadic range decrement, V	6.401	77.1	95.8	18.51	0.24	0.906 (0.826–0.986)	** < 0.001**
Smooth-pursuit range decrement, H	9.077	51.4	100	+ ∞	0.48	0.722 (0.591–0.853)	**0.004**
Smooth-pursuit range decrement, V	6.491	60	95.8	14.29	0.42	0.786 (0.667–0.904)	** < 0.001**
In less-affected eyes							
Saccadic range decrement, H	5.956	60	83.3	3.59	0.48	0.69 (0.553–0.827)	**0.014**
Saccadic range decrement, V	7.264	62.9	95.8	14.98	0.39	0.739 (0.607–0.871)	**0.002**
Smooth-pursuit range decrement, H	9.98	28.6	100	+ ∞	0.71	0.498 (0.348–0.648)	0.982
Smooth-pursuit range decrement, V	4.939	42.9	87.5	3.43	0.65	0.546 (0.397–0.696)	0.547
Generalized MG							
In more-affected eyes							
Saccadic range decrement, H	7.522	81.8	100	+ ∞	0.18	0.894 (0.723–1)	** < 0.001**
Saccadic range decrement, V	5.818	90.9	91.7	10.95	0.1	0.924 (0.801–1)	** < 0.001**
Smooth-pursuit range decrement, H	3.877	72.7	75	2.908	0.36	0.742 (0.539–0.946)	**0.023**
Smooth-pursuit range decrement, V	5.003	72.7	87.5	5.82	0.31	0.833 (0.668–0.999)	**0.002**
In less-affected eyes							
Saccadic range decrement, H	6.835	72.7	95.8	17.31	0.28	0.913 (0.806–1)	** < 0.001**
Saccadic range decrement, V	5.426	72.7	87.5	5.82	0.31	0.826 (0.655–0.996)	**0.002**
Smooth-pursuit range decrement, H	21.138	18.2	100	+ ∞	0.82	0.439 (0.2–0.679)	0.57
Smooth-pursuit range decrement, V	12.006	36.4	100	+ ∞	0.64	0.689 (0.483–0.896)	0.076

Statistical significance was calculated using ROC analysis at the significance level of 0.05

*AUC* area under the receiver operating characteristic curve, *CI* confidence interval; *H* horizontal; *LR* likelihood ratio; *V* vertical

In addition, Pearson correlation analyses showed no correlation between the magnitudes of decrements and the demographic features of age, sex, and symptom duration (*p* > 0.05).

## Discussion

We aimed to explore whether the oculomotor decrements after repetitive saccadic and smooth-pursuit eye movements using VOG could be used to detect MG. The oculomotor tests using VOG enabled us to quantify objectively the overall pattern of involved muscles of oculomotor fatigability in our patient group. Identifying saccadic decrements (> 7.2% for horizontal and > 6.4% for vertical) in more-affected eyes allowed us to differentiate between the MG and HC groups with a high sensitivity (76.1% for horizontal and 78.3% for vertical) and specificity (100% for horizontal and 95.8% for vertical). Even in less-affected eyes, saccadic decrements allowed differentiation between MG patients and HCs with a sensitivity of 54.3% and a specificity of 95.8%. The smooth-pursuit range decrements also demonstrated a relatively good diagnostic power to differentiate between MG patients and HCs. Hereby, we demonstrated the diagnostic utility of quantifying the oculomotor decrements after repetitive saccades and smooth pursuits to reflect sensitively the oculomotor fatigue found in MG patients.

Saccadic fatigability in normal subjects has been reported as an increase in motion time and a decrease in amplitude appearing as undershooting or hypometria during 20 cycles of repetitive saccadic eye movements [34]. Another study using bandwidth photodiode analysis described oculomotor fatigue following 30 sequential saccades of 50^°^ magnitude as low-velocity, long-duration, and non-main sequence saccades [35]. A recent case–control study applying VOG to 18 patients with OMG and 50 HCs revealed a substantial increase in both horizontal inward and vertical downward latency and a significant reduction in the mean vertical downward amplitude following repetitive saccadic movements in MG patients compared to the control group [25]. Our preliminary results demonstrate that VOG analysis of two OMG patients showed a substantial decrease in gain of horizontal smooth pursuits following 50 cycles of pursuit in the range of 20°. In a prospective cohort study, ocular muscle fatigability was systematically investigated by asking participants to avert their gaze for 60 s in two horizontal and four oblique directions. Accordingly, diplopia was observed in 64% of 144 MG patients and 0% of 20 healthy controls [36]. A paradoxical finding in myasthenic patients indicating normal or increased saccadic velocity in the presence of smooth-pursuit deficits has been well described in the literature [26, 37–39]. Although several studies on oculomotor fatigability in normal and MG patients have been published [25], this variability of saccadic velocity requires validity and reliability testing for application to diagnosis of MG.

Decrements in the saccadic range seem to provide greater discriminatory power than pursuit decrements, despite having similar best cutoff values in ROC curve analysis (Fig. 3). The respective sensitivity, specificity, and AUC values were 78.3%, 95.8%, and 0.91 (95% CI, 0.841–0.979) with an optimal cutoff value of 6.401% for decrement in vertical saccadic ranges and 60.9%, 95.8%, and 0.797 (95% CI, 0.694–0.9) with an optimal cutoff value of 6.399% for vertical smooth pursuit. The stimulus for a saccade is the target position with respect to the fovea, whereas the stimulus for a smooth pursuit is the target velocity relative to the retina. In humans, the differential response of eye movements has been explained based on the composition of muscle fibers recruited among electrically designated fast- and slow-twitching types [40]. Ocular muscles contain multiply innervated fibers that function similarly to slow fibers in addition to singly innervated fibers classified based on contraction speed and fatigue resistance [41]. Correlative anatomical, molecular, and physiological studies of ocular muscles have revealed that all motoneurons and all ocular muscle fiber types participate in all eye-movement classes and support the heterogeneity of ocular muscle fiber types as a consequence of their recruitment at specific eye positions requiring a range of contractile and fatigability properties [41]. Therefore, based on the physiologic characteristics of the ocular muscles, oculomotor fatigability may be observed similarly in saccadic and smooth-pursuit movements.

In previous studies, voluntary saccades were studied by electro-oculography in small numbers of MG patients, with analyses mostly focusing on saccadic velocity and accuracy [35, 42]. Compared to control subjects, the velocities of saccades were preserved or increased during the initial phase or during small saccades (1°–3°), while the amplitudes were reduced [37]. In a study that tracked the small-range movement of both eyes in a 60-year-old MG patient, researchers discovered a decrease in amplitude of smooth pursuits in the right eye with conjunctive velocity components and hypermetric saccades with disjunctive velocity components [37]. Another study found that MG patients experienced a significant increase in saccadic peak velocity at 10^°^ but not at 20^°^, with no difference in saccadic amplitude and with a reduced smooth-pursuit amplitude compared to controls [26]. In this context, the disconjugacy of the velocity profiles of the initial saccade components was caused by impairment of all types of muscle fibers, whereas abnormally fast saccade velocities and saccadic hypermetria with dynamic overshoot were explained by central upregulation of saccadic gain. In MG patients, saccadic fatigue can produce overlapping saccades with high-frequency saccadic bursts separated by long pauses and glissades in which the high-frequency bursts are much shorter than appropriate for the size of the intended saccades. Low-velocity, long-duration, non-main sequence saccades in MG patients compared to normal subjects were also reported [35]. Another study reported that the maximum velocities of 20° and 40° saccades in patients with MG were not significantly different from those in HCs, while patients with other types of ophthalmoplegia showed significantly decreased maximum velocities. A recent small pilot study demonstrated greater saccadic peak velocity in MG patients compared to HCs [26]. Therefore, relatively preserved saccadic velocities in MG patients suggest that twitching muscle fibers generating rapid movements during initial saccades are spared [42]. In the current fatigue quantifications, the diagnostic value in the vertical plane of both saccades and smooth pursuits seems to be superior than that in the horizontal plane in distinguishing MG patients from HCs. MG is characterized by bilateral, fluctuating, and combined extraocular muscle weakness, suggesting that fatigable resistance is not the same in the two movement planes. Several studies have reported the elevating and adducting muscles to be more frequently involved than the depressing and abducting muscles [43, 44]. Muscles that have fewer spindles [45] but greater bulk have a tendency to be more easily fatigued than other muscles [46].

As in previous studies [20, 29, 47, 48], in the current study, the positivity of the ancillary tests, such as the AChR antibody test (60% *vs.* 100%; *p* = 0.013, Mann–Whitney *U* test), RNS test (31.4% *vs.* 72.7%, *p* = 0.016), and edrophonium test (45.7% *vs.* 81.8%, *p* = 0.038) were significantly lower in the OMG subgroup than that in the GMG subgroup (Table 1). We found that the sensitivities for detecting OMG with decrements of oculomotor ranges after repetitive movements were > 70% in saccadic decrement and > 50% in pursuit decrement and were equally high in both ocular and generalized MG, while the traditional diagnostic procedures suffer reduced sensitivity (from 30%–60%) in OMG [16, 49]. Furthermore, in OMG (*n = *35), the vertical saccadic decrement was higher than the cutoff in 79.17% (19/24) of patients with a normal RNS and in 85.71% (12/14) of seronegative patients. In GMG (*n = *11), the vertical saccadic decrement was higher than the cutoff in 67.7% (2/3) of patients who had a normal RNS result. Thus, the decrement of vertical saccadic ranges allowed us to identify myasthenic oculomotor fatigability even in subjects with negative RNS and serologic antibody tests. The results from the current cross-sectional study suggest that quantifying oculomotor fatigability based on the range of decrements after repetitive eye movements can provide a differential diagnosis of MG from HCs because this test directly assesses the fatigability of the ocular muscles.

Limitations of this study include that our patients from a tertiary referral center may not reflect the total MG population due to a referral bias. In addition, we focused on the decrements of oculomotor range and did not consider other saccadic and pursuit characteristics. These should be analyzed in further studies. In addition, further prospective studies are warranted to establish the definitive value of our model and to validate its use in clinical practice for differentiating patients with MG from those with other neurologic disorders. AChR antibody seronegative patients were tested with serum auto-antibodies against MuSK by radioimmunological assay, and all were negative. However, these patients were not analyzed with a cell-based assay to increase detection of antibodies. Therefore, we also need to establish VOG-based oculomotor decrements in patients with MuSK-MG and in those with other auto-antibodies.

This cross-sectional prospective study provides class III evidence for VOG-based quantification of saccadic and smooth-pursuit fatigability to distinguish between MG patients and HCs. This method particularly facilitates diagnosis in patients with isolated ocular involvement, negative serology, and conventional RNS studies. VOG analysis can provide direct evidence of oculomotor fatigability and has unique advantages as a reliable, safe, and simple test. This highly sensitive and specific diagnostic tool could have a large clinical impact for earlier diagnosis, resulting in earlier initiation of appropriate treatments and improved patient care and quality of life.

## Data Availability

All individual data of the participants that underlie the results reported in this article will be available after de-identification (manuscript, tables, and figures).
